# Surgically induced scleritis after pars plana vitrectomy or scleral buckling surgery: a 12-year audit

**DOI:** 10.1038/s41433-025-03803-4

**Published:** 2025-04-16

**Authors:** Antony Raharja, Neil Shah, Syed Ahmed, Siegfried Karl Wagner, Narciss Okhravi, James Bainbridge

**Affiliations:** 1 Moorfields Eye Hospital, London, UK; 2 UCL Institute of Ophthalmology, London, UK

**Keywords:** Pain, Risk factors, Scleral diseases

## Abstract

**Background:**

Surgically induced scleritis is a recognised adverse consequence of retinal surgery but its incidence, clinical features and outcomes are poorly defined.

**Methods:**

This retrospective case series included all cases of surgically induced scleritis following pars plana vitrectomy (PPV) or scleral buckling surgery (SB) identified at Moorfields Eye Hospital, London, UK between September 2012 and June 2024. Those with a previous history of scleritis were excluded. The incidence was estimated using identified cases and the total number of PPV and SB performed during the same period. Clinical characteristics, management and outcomes were described.

**Results:**

Forty cases of surgically induced scleritis were identified: 23 post-PPV and 17 post-SB. The estimated annual incidence was 5.0 cases (95% CI: 3.4–7.5) per 100,000 PPV and 45.0 cases (95% CI: 24.4–82.5) per 100,000 SB. Scleritis was non-necrotising in 62.5% cases and developed following multiple retinal surgeries in 45% cases. Post-PPV scleritis presented earlier (3.6 weeks vs. 33.4 weeks, *p* = 0.013), but appeared less likely to be necrotising (26% vs. 47%, *p* = 0.20) than SB scleritis. Oral NSAIDs were sufficient to control scleritis in 33 (82.5%) cases. Systemic steroids or immunomodulatory therapy were used more frequently in SB scleritis than post-PPV scleritis (29.4% vs. 8.7%, *p* = 0.11). The scleral explant was removed in 59% cases of SB scleritis. A scleral patch graft was applied in 5% cases. With treatment, visual acuity at presentation was maintained in 80% of the cases and the globe preserved in all.

**Conclusions:**

Surgically induced scleritis following retinal surgery is rare. Scleritis associated with scleral buckling appears to require more aggressive medical and surgical intervention than scleritis following pars plana vitrectomy.

## Introduction

Surgically induced necrotising scleritis (SINS) is a rare and potentially devastating consequence of ocular surgery [1, 2]. The landmark study in 1992 by O’Donoghue et al. highlighted the key features of SINS as necrotising inflammation occurring at surgical wound sites many months (mean interval of nine months) after multiple ocular procedures [1]. An underlying systemic disease was noted in 63% of patients.

The relevance of these findings to current vitreoretinal surgery may be limited since in only a small proportion (seven eyes, 13%) of this heterogenous cohort was inflammation incited by retinal procedures. Subsequent reports have described clinical phenotypes that differ from the classic SINS as originally described [3–5]. For instance, Morley et al. reported three cases of necrotising scleritis presenting much earlier (2–6 weeks) following 20-gauge (20 G) pars plana vitrectomy (PPV) and did not find an underlying vasculitis [3]. Infective scleritis had also been reported within a week following 20 G PPV and scleral buckling surgery (SB) [6]. Furthermore, vitreoretinal practice has since progressed towards smaller gauge instruments for PPV and away from encirclement for SB. Despite a substantial body of literature on the most severe and dramatic presentation of post-surgical scleritis i.e. necrotising scleritis, information on outcomes of surgically induced non-necrotising or ‘diffuse’ scleritis (SIDS) is limited [5].

Updated evidence on scleritis following vitreoretinal surgeries could help to further characterise this uncommon adverse event and optimise its management. In this study, we sought to determine the incidence of surgically induced scleritis following PPV or SB surgery in a large tertiary eye centre over a 12-year period. We describe the clinical features, management approaches and outcomes.

## Methods

This is a retrospective study of all patients diagnosed with surgically induced scleritis after PPV or SB between 01/09/2012 and 05/06/2024 in Moorfields Eye Hospital NHS Foundation Trust, London, UK. Cases were identified from the hospital electronic medical record database, OpenEyes (Across Health, Ghent, Belgium) by cross-referencing all coded vitrectomy and scleral buckle procedures with free text search terms of “scleritis”. Individual patient records were reviewed (by AR, NS, SA) to determine eligibility for this study and for data collection. We included all patients who developed surgically induced scleritis after PPV or SB. We excluded episcleritis, suture-related granuloma and eyes with a previous history of scleritis prior to PPV or scleral buckle. Those who only received anterior vitrectomy without full pars plana vitrectomy were also excluded.

Scleritis was diagnosed by the treating clinicians and subclassified as anterior (diffuse, nodular or necrotising) and/or posterior according to Watson and Hayreh [7]. Surgically induced anterior scleritis was defined as scleral oedema with injection of deep episcleral vessels without blanching by 2.5–10% topical phenylephrine and must involve the sclerostomy or buckle site [1, 2]. Necrotising subtype was diagnosed based on the clinical finding of scleromalacia or anterior segment fluorescein angiography result [8]. Posterior scleritis was diagnosed with a combination of clinical and ultrasonographic findings [9].

In this study, we described patient demographics, surgical factors, clinical features, management, and outcomes of all cases seen in our institution during the study period [10]. The cumulative incidence (incidence proportion) of surgically induced scleritis post-PPV and -SB was separately calculated using the identified cases (excluding those whose surgery was performed elsewhere or outside the study period) and the total number of PPV and SB procedures performed in our institution during the 141-month study period. The annual incidence was then estimated, with the 95% confidence interval calculated using the Wilson/Brown method. For those who underwent both PPV and SB (combined or sequential), the inciting surgery was determined by the treating clinicians based on the site of scleral inflammation, temporal association to onset of scleritis and the response to removal of the scleral explant where relevant. Such cases were counted only once for incidence estimation.

Continuous variables were reported using median (interquartile range, IQR) whereas categorical parameters were described in numbers (percentage, %). Snellen visual acuity (VA) measurements were converted to logarithm of the minimum angle of resolution (logMAR), including counting finger (2.0 logMAR), hand movement (2.3 logMAR), light perception (2.7 logMAR) and no light perception (3.0 logMAR) [11]. The Mann–Whitney U test or Fisher’s exact test was used to compared two groups, as appropriate. All statistical tests were carried out using GraphPad Prism, version 10 (GraphPad software, San Diego, CA, USA). This study was registered as an audit, received institutional review board approval (audit number 1486) and was conducted in accordance with principles stated in the Declaration of Helsinki.

## Results

### Incidence estimation

During the 141-month study period, there were 40 cases of surgically induced scleritis: 23 cases after PPV and 17 cases after SB. During the same period, there were 38,935 PPV in 28,439 patients and 1893 SB from 1595 patients conducted at all sites of Moorfields Eye Hospital NHS Foundation Trust. Seven cases of scleritis post-SB were excluded for incidence calculation as the inciting SB surgery was performed elsewhere (*n* = 4) or outside the study period (*n* = 3). Hence, the estimated annual incidence of surgically induced scleritis in our institution was 5.0 cases (95% CI: 3.4–7.5) per 100,000 PPV and 45.0 cases (95% CI: 24.4–82.5) per 100,000 SB. When considering only necrotising scleritis cases, the estimated annual incidence was 1.3 (95% CI: 0.60–2.86) cases per 100,000 PPV and 9.0 (1.6–32.7) cases per 100,000 SB.

### Surgically induced scleritis after PPV

Of the 23 patients (23 eyes) who developed scleritis after PPV, 16 (70%) were male, 12 (52%) were of white Caucasian ethnicity and their median age was 54 years (IQR 39–66) (Table 1). A history of significant ocular trauma was noted in two (8.7%) eyes. Scleritis developed after a single PPV operation in 11 (48%) eyes including three (13%) with prior phacoemulsification cataract surgeries. The remaining 12 (52%) eyes had multiple retinal procedures with a median of three PPV (IQR 2.25–4.75), including two eyes with previous SB, ten combined phacovitrectomy and one Baerveldt glaucoma implant. None had extracapsular cataract surgery through limbal incision or pterygium surgery, which are two leading causes of SINS in previous literature [1, 12].

**Table 1:** Characteristics of patients who developed scleritis following pars plana vitrecomy (PPV) and scleral buckle (SB) surgery.

	Scleritis following pars plana vitrectomy (*n* = 23 patients (23 eyes))	Scleritis following scleral buckle (*n* = 16 patients (17 eyes))
Age, year	54.0 (39.0–66.0)	38 (30.5–57.3)
Male	16 (70%)	7 (44%)
Ethnicity		
White	12 (52%)	5 (31%)
Black	4 (17%)	1 (6.3%)
Asian	4 (17%)	5 (31%)
Mixed, others or unknown	3 (13%)	5 (31%)
Systemic autoimmune or connective tissue disease	3 (13%)	2 (13%)
Previous ocular comorbidities		
Scleritis	0 (0%)	0 (0%)
Uveitis	4 (17%)	0 (0%)
Glaucoma	1 (4.3%)	0 (0%)
Ocular trauma	2 (8.7%)	3 (18%)
Retinal surgery preceding onset of scleritis		
Single retina surgery only	11 (48%)	11 (65%)
Multiple retinal surgeries	12 (52%)	6 (35%)

Data were presented as *n* (%) or median number (IQR, interquartile range).

Indications for PPV were retinal detachment (RD) (9 cases, 39%), removal of silicone oil post-RD repair (6 cases, 26%), scleral-fixated intraocular lens (IOL) implantation (3 cases, 13%), full-thickness macula hole (2 cases, 8.7%), macula proliferative vitreoretinopathy (1 case, 4.3%), iris-fixated IOL implantation (1 case, 4.3%), and intraocular foreign body removal (1 case, 4.3%). The vitrectomy gauge used were 23 G in 19 (83%) cases, and 25 G in 4 (17%) cases. Sclerostomy ports were sutured in 15 (65%) cases (Table 2).

**Table 2:** Operative characteristics of inciting surgeries.

	Scleritis following pars plana vitrectomy (*n* = 23 eyes)	Scleritis following scleral buckle (*n* = 17 eyes)
Vitrectomy gauge or scleral explant	23G: 19 (83%)	Encircling SB: 8 (47%)
	25G: 4 (17%)	Segmental SB with silicone tire: 7 (41%)
		Sponge: 2 (12%)
Intraocular tamponade	Air: 5 (22%)	None: 10 (59%)
	SF6: 4 (17%)	SF6: 2 (12%)
	C2F6: 1 (4.3%)	Unknown:5 (29%)
	C3F8: 4 (17%)	
	SiO2: 3 (13%)	
	BSS: 6 (26%)	
Use of sutures	15 (65%)	23 (100%)
Surgeon grade	Registrar: 1	Fellow: 10
	Fellow: 15	Consultant: 2
	Consultant: 6	Unknown: 5

Scleritis presented 3.6 weeks (IQR 2.1–51.7) after PPV, with BCVA of 0.69 logMAR (IQR 0.30–1.35) (Table 3). Scleritis was frequently associated with mild anterior uveitis of at least grade 0.5+ anterior chamber cells (10 cases, 43%), occasionally with ocular hypertension exceeding 21 mmHg (2 cases, 8.7%) and rarely with vitritis, cystoid macular oedema (CMO) or chorioretinal folds (1 case, 4.3%). None had documented interstitial or peripheral ulcerative keratitis.

**Table 3:** Clinical features, management and outcomes of surgically induced scleritis following PPV or SB.

	Scleritis following pars plana vitrectomy (*n* = 23 patients (23 eyes))	Scleritis following scleral buckle (*n* = 16 patients (17 eyes))
Time interval between surgery and scleritis, weeks	3.6 (2.1–51.7)	33.4 (5.7–756.7)
Scleritis subtypes		
Anterior diffuse	16 (70%)	8 (47%)
Anterior nodular	1^a^ (4.3%)	0 (0%)
Anterior necrotising	6 (26%)	8 (47%)
Posterior	2 (8.7%)	1 (6%)
Aetiology		
Non-infective scleritis	21 (91%)	15 (94%)
Infective scleritis	2 (8.7%)	1 (6%)
Treatment		
NSAIDs	20 (87%)	11 (65%)
Systemic steroids	2 (8.7%)	5 (17%)
Immunomodulatory therapy	1 (4.3%)	2 (12%)
Surgical intervention	2 (8.7%)	10 (59%)
Duration of follow-up, months	15 (5–69)	8 (5–21)
Best corrected visual acuity (BCVA), logMAR		
At presentation	0.69 (0.30–1.35)	0.54 (0.08–1.36)
At final follow-up	0.54 (0.08–1.61)	0.48 (0.10–1.00)
Change in vision^b^	−0.18 (−0.40–0.13)	0.00 (−0.20–0.08)
Scleritis recurrence	4 (17%)	5 (29%)

^a^There were three cases of nodular scleritis but two developed features of necrotising scleritis and were reclassified.

^b^negative value indicates improvement in vision at final follow-up compared to presenting BCVA at onset of scleritis.

Scleritis following PPV was anterior non-necrotising in 17 (74%) eyes and necrotising in 6 (26%) eyes. Anterior non-necrotising scleritis were of diffuse subtype in 16 eyes and nodular subtype in one eye. Two (7.8%) eyes with anterior necrotising scleritis also exhibited posterior scleritis – one at the time of anterior scleritis (with findings of serous retinal detachment, posterior coat thickening and T-sign on B-sign), and one at recurrence.

Oral NSAIDs in combination with topical steroids and/or NSAIDs were sufficient for post-PPV anterior non-necrotising scleritis cases. Flurbiprofen was the most commonly used oral NSAIDs with a median daily dose of 150 mg (IQR 150–300) and treatment duration of 2 weeks (IQR 2–4) in the post-PPV scleritis group.

Of the six eyes with necrotising scleritis following PPV, two required systemic steroids or immunomodulatory therapy (IMT); these two cases were microbiology-proven infective scleritis. One case required surgical removal of scleral-fixated IOL and the associated expanded polytetrafluoroethylene (GORE-TEX) suture. The remaining three cases initially presented as diffuse anterior scleritis, with scleromalacia being subsequently recognised during follow-up examinations. In these cases, scleritis responded adequately to oral NSAIDs and was deemed not to require further escalation of treatment by our uveitis team. Delayed recognition of necrotising scleritis was also noted in the landmark study by O’Donoghue et al., in which less than 60% of the SINS were identified at the initial clinical evaluation, with the remainder recognised during subsequent follow-up examinations or with anterior segment fluorescein angiography [1].

The aetiology of surgically induced scleritis post PPV was non-infective in 21 (91%) cases. There were two (8.7%) cases of microbiology-proven infective scleritis. Subconjunctival abscess was a feature in both cases, albeit not an initial presenting feature in one of the two cases. The causative pathogens were *Haemophilus Influenza* in one case, and mixed organisms in the other (*Pseudomonas aeruginosa* and *Aspergillus fumigatus*). Oral NSAIDs were insufficient to control inflammation in both cases. The former required oral prednisolone, moxifloxacin and surgical intervention for debridement and application of scleral patch graft. Oral prednisolone was tapered over 19 months. The latter initially responded to oral prednisolone, before deteriorating when dose was reduced. Treatment was escalated to intravenous methylprednisolone pulse therapy and ciclosporin which continued for another month before the diagnosis of co-existing fungal infection.

At 15 months’ follow-up (IQR 5–69), the final BCVA in the post-PPV scleritis cohort was 0.54 (IQR 0.08–1.61). The median change in BCVA at final follow-up from diagnosis was −0.18 (−0.40–0.13), indicating most eyes maintain or gain vision with treatment. Vision loss of at least one line was seen in five (22%) eyes. Four (17%) cases had one episode of scleritis recurrence. All recurrences had the same morphology as the initial presentation except in one eye (suspected posterior scleritis based on mildly reduced vision and T-sign on ultrasound B-scan). These responded to oral NSAIDs and did not require escalation of treatment to immunomodulatory treatment.

An underlying connective tissue or autoimmune disease was uncommon (13%) in the post-PPV scleritis cases; two patients had Marfan syndrome, and one had ulcerative colitis. Systemic vasculitis or rheumatoid disease was not detected in the five (22%) patients who underwent laboratory and radiographic work-up.

### Surgically induced scleritis after SB

Of the 16 patients (17 eyes) who developed scleritis after SB, seven (44%) were male, five (31%) were of white Caucasians ethnicity and their median age was 38 years (IQR 31–57) (Table 1). A history of significant ocular trauma was noted in three (18%) eyes. Scleritis developed after a single SB procedure in 11 (65%) eyes. The remaining six (35%) eyes had multiple retinal procedures including previous SB (2 cases), combined PPV-SB procedures (2 cases), and subsequent PPVs after the initial SB (2 cases). Removal of suture triggered development of scleritis in one eye.

The scleral explants used were encircling bands (47%) in eight eyes (including four with an additional silicone tire segment, and one with an additional sponge), segmental silicone tire buckle in seven (41%) cases (including one with additional sponge), and sponge in two (12%) cases (Table 2).

Scleritis presented 33.4 weeks (IQR 5.7–756.7) after SB, with BCVA of 0.54 logMAR (IQR 0.08–1.36) (Table 3). Buckle extrusion was a presenting feature in seven (41%) cases. The latency period between surgery and scleritis onset is significantly longer for post-SB than post-PPV (*p* = 0.013).

Scleritis following SB was anterior non-necrotising (diffuse subtype) in eight (47%) eyes, necrotising in eight (47%) eyes and posterior in one (6%) eye. Scleritis after SB was frequently associated with mild anterior uveitis (4 cases, 24%) and rarely with ocular hypertension or vitritis (1 case, 6%). No keratitis, CMO or chorioretinal folds were reported.

The aetiology of surgically induced scleritis post SB was non-infective in 16 (94%) cases. There was only one (6%) microbiology-proven infective scleritis caused by *Staphylococcus aureus*.

Treatment included oral NSAIDs in the majority of cases; Flurbiprofen was most commonly used with a median daily dose of 300 mg (IQR 275–300) and treatment duration of 3 weeks (IQR 2–4) in the post-SB scleritis group. Five (29%) cases of necrotising scleritis required systemic steroids and two (12%) required immunomodulatory therapy (IMT). Oral prednisolone was successfully tapered over 6–9 weeks in three cases. One patient received long-term IMT due to subsequent diagnosis of a systemic vasculitis and the other received mycophenolate mofetil for 2.5 years before being successfully weaned off following the removal of scleral explant.

Oral moxifloxacin was used in three cases in which infection was suspected due to finding of eyelid erythema, swelling and mucopurulent discharge. Wound swabs and scleral explants were sent for microscopy, culture and sensitivity but only one (6%) showed positive culture with staphylococcus aureus from both conjunctival swab and scleral explant. One other case showed gram negative rods on microscopy but culture was negative.

The scleral explant was removed in 10 (59%) cases, including one (6%) requiring a scleral patch graft. One patient declined removal of the scleral explant despite recurrence of scleritis due to concerns of re-detachment. Amongst those who underwent removal of scleral explant, there was no recurrent retinal detachment.

At 8 months’ follow-up (IQR 5–21), the final BCVA was 0.48 (IQR 0.10–1.00). Scleritis recurred in 5 (29%) cases. The median change in BCVA from diagnosis to final follow-up was 0.00 (−0.20–0.08), with majority of patients maintain or gaining vision with treatment. Three (18%) patients lost at least one line of vision.

Prior to surgery, one patient was known to have multiple sclerosis. Of the six (35%) patients who underwent laboratory and radiographic work-up, one was found to have a systemic vasculitis (granulomatosis with polyangiitis). Taken together, an underlying autoimmune disease was uncommon (12.5%).

## Discussion

This study provides contemporary data on the clinical features, management, and outcomes of surgically induced scleritis following retinal surgery. To our knowledge, this is the largest case series reporting surgically induced scleritis post-PPV and post-SB. The data provide an estimate of its incidence and confirm that this post-operative complication is rare.

The estimated annual incidence of scleritis following PPV and SB in our cohort was low especially following PPV: 5.0 cases (95% CI: 3.4–7.5) per 100,000 PPV and 45.0 cases (95% CI: 24.4–82.5) per 100,000 SB. Nevertheless, the risk of scleritis following retinal surgery appears higher than the baseline incidence of scleritis in the general population in the United Kingdom (2.79 cases [95% CI 2.19–3.39] per 100,000 person-year), suggesting that retinal surgery is likely contributory [13]. Of note, the risk of scleritis following SB was nine times higher than that following PPV, and 16 times higher than in general population.

Overall, scleritis following retinal surgery was non-necrotising in 62.5% cases. The mainstay of treatment in our cohort was oral NSAIDs in 82.5% cases. Systemic steroids and immunomodulatory therapy were required in 17.5% and 7.5% cases respectively. Outcomes were generally good, with vision maintained in 80% of the cases and the globe preserved in all patients within our cohort.

Surgically induced scleritis following retinal surgery demonstrates clinical heterogeneity and its clinical features varies with the causative surgery and underlying aetiology. Scleritis following SB presented later (33.4 weeks vs. 3.6 weeks, *p* = 0.013) and appeared more likely to be necrotising (47% vs. 26%, *p* = 0.20). Systemic steroids or immunomodulatory therapy (29.4% vs. 8.7%, *p* = 0.11) and surgical intervention (59% vs. 8.7%, *p* = 0.001) were also required more often in scleritis post-SB than post-PPV. The underlying aetiology in most cases was non-infective. Despite being relatively less common, in keeping with published literature [12], post-surgical infective scleritis was more severe and required more aggressive medical or surgical treatment. For instance, in the post-PPV scleritis cohort, oral NSAIDs were sufficient for non-infective cases whereas the two infective scleritis cases required systemic steroids or IMT.

The exact pathogenesis of non-infective surgically induced scleritis is unclear. Necrotising scleritis may be more common following SB than PPV because SB is primarily an extraocular procedure with most of the surgical manoeuvres carried out at the level of sclera. Given the improvement in scleritis following removal of scleral explant, it is thought that scleral buckling especially encirclement induces scleritis via mechanical compression and ischaemia [1]. However, experimental occlusion of vascular supply to sclera in rabbits and excessive cautery in human sclera has not been shown to cause inflammatory scleral necrosis [14]. Thus, ischaemia alone appears insufficient and other factors such as abnormal response to surgical trauma, increased collagenase action and immune complex deposition may be involved [3, 15].

The rate of necrotising disease was lower in our cohort than in previous case series [1, 12]. For instance, O’Donoghue et al. [1] reported 96% of post-surgical scleritis to be necrotising compared to 37.5% in the current study. This stark difference could be due to the inherent differences in the tendency of different ocular procedures to cause necrotising disease. In the previous study, extracapsular cataract extraction through a limbal incision, a well-recognised trigger for SINS [15], was the most common surgery implicated. Only 13% of the cases were related to retinal procedures. Furthermore, there could be selection bias in previous case series which sampled cases from specialised scleritis [1] or uveitis [3] clinics. We harnessed our robust electronic medical record database to systematically identify all relevant cases of surgically induced scleritis after PPV or SB. This enabled both necrotising and less severe non-necrotising forms to be captured in this retrospective study, reducing the risk of selection bias. The lack of use of anterior segment fluorescein angiography in routine clinical practice especially in milder cases may also explain the low rate of necrotising disease identified in our cohort. Another potential explanation is a true reduction in the incidence of post-surgical necrotising scleritis with the progress in VR surgery techniques.

Due to the lack of matched controls, we were unable to conclusively identify surgery-related risk factors. However, 23 G PPV and encircling bands were the most implicated in our cohort of surgically induced scleritis post PPV (83%) and post SB (47%) respectively, despite the latter being uncommonly used in our institution. Although not entirely representative of the current study, the Moorfields Buckle Study indicated that encirclement accounted for only 5.7% of all SB carried out for primary RD repair between 2004 and 2022. [16].

The rate of underlying autoimmune disease in our cohort was found to be low. Pre-existing connective tissue or autoimmune disease was noted in 10% of the cohort. This study was limited in investigating the utility of systemic work-up for surgically induced scleritis following retina surgery, as systemic work-up was not routinely done in all patients and appeared to be reserved to those with severe or recurrent disease. Of the eleven (28%) patients who received systemic work-up, a systemic vasculitis was found in one patient. If work-up had been offered to all patients including those with milder non-necrotising forms, the yield of systemic work-up may have been lower (assuming a positive test is less likely in those with milder disease). Our study finding is in keeping with previous literature review in 2013 which found low rates of underlying autoimmune disease in patients with necrotising scleritis following PPV (1 of 8; 13%), SB (2 of 36; 5.6%) and other ocular surgeries except cataract extraction through limbal incision (24 of 56; 43%) [12].

The main limitation of this study is its retrospective nature. Although a suitable study design to characterise rare adverse events, its ability to accurately estimate incidence relies on accurate record keeping. The extent of scleritis (i.e. number of quadrants involved) was not always clearly quantified. Additionally, as onset of scleritis may be months or even years after the surgery, this long interval time may introduce bias and underestimate incidence if follow-up is insufficient. The influence of surgically induced scleritis on vision may not be fully appreciated in this case series due to different retinal pathologies confounding the pre- and post-surgery vision, and that scleritis occurred at different time points during post-operative visual recovery. Nevertheless, this study provides reassurance that vision can be maintained with appropriate management of this complication.

In summary, surgically induced scleritis is a rare consequence of retinal surgery. This study highlights key differences between scleritis post-PPV and post-SB. In addition to identifying and treating any infection, management of inflammation using NSAIDs, immunosuppressive therapy, and surgical intervention can be associated with favourable visual outcomes.

## Summary

### What was known before:

- Surgically induced necrotising scleritis (SINS) is a recognised consequence of ocular surgery including retinal surgery.
- The incidence, clinical characteristics, and outcomes of surgically induced scleritis following retinal surgery are poorly defined.

### What this study adds:

- The estimated annual incidence of surgically induced scleritis was 5.0 cases (95% CI: 3.4–7.5) per 100,000 PPV and 45.0 cases (95% CI: 24.4–82.5) per 100,000 SB.
- Scleritis following retinal surgery was non-necrotising in 62.5% cases and an underlying autoimmune or connective tissue disease was uncommon (13%).
- Compared to post-PPV cases, scleritis following scleral buckling surgery presented later, but were more likely to require systemic steroids, immunomodulatory therapy or surgical intervention.

## Data Availability

Anonymised data presented in this study are available on reasonable request from corresponding author and approval by Moorfields Eye Hospital NHS Foundation Trust.
